# Effects and interactions of dietary lysine and apparent nitrogen corrected metabolizable energy on yellow-feathered broiler breeder hens

**DOI:** 10.1186/s40104-024-01095-4

**Published:** 2024-11-01

**Authors:** Yibing Wang, Qin Wang, Xiangtian Yao, Zhongyong Gou, Xiajing Lin, Qili Luo, Shouqun Jiang

**Affiliations:** Institute of Animal Science, Guangdong Academy of Agricultural Sciences, State Key Laboratory of Swine and Poultry Breeding Industry, Key Laboratory of Animal Nutrition and Feed Science in South China, Ministry of Agriculture and Rural Affairs, Guangdong Provincial Key Laboratory of Animal Breeding and Nutrition, No.1 Dafeng Street 1, Wushan, Tianhe District, Guangzhou, 510640 China

**Keywords:** Breeder hens, Egg quality, Lysine, Metabolizable energy, Productive performance, Reproductive traits

## Abstract

**Background:**

Dietary lysine and apparent nitrogen-corrected metabolizable energy (AMEn) are two key variables affecting the production of breeder hens. In this study, the effects and interactions of lysine and AMEn on yellow-feathered broiler breeder hens were investigated. A total of 720 30-week-old breeder hens were fed in a 5 (lysine: 0.56%, 0.68%, 0.80%, 0.92%, and 1.04%) × 2 (AMEn: 11.50 and 11.10 MJ/kg) factorial arrangement for 12 weeks. The productive performance, reproductive traits, biochemical variables of breeder hens, the amino acid concentration and quality of eggs, and the growth performance of offspring broilers were determined.

**Result:**

(1) Dietary lysine had quadratic effects (*P* < 0.05) on laying rate, average daily egg mass and feed intake/egg mass of breeder hens; birds with 11.50 MJ/kg AMEn (high AMEn) had higher (*P* < 0.05) BW than those with 11.10 MJ/kg AMEn (low AMEn); (2) dietary lysine significantly affected on the relative ovarian weight (quadratic and linear), and numbers of large yellow follicles (LYF, quadratic); birds with high AMEn had longer fallopian tube and more LYF than those with low AMEn (*P* < 0.05); (3) dietary lysine had significant effects (linear and quadratic) on eggshell thickness and shell strength of eggs from breeder hens; birds with high AMEn had thinner eggshells and deeper yolk color than those with low AMEn (*P* < 0.05); (4) there were higher (*P* < 0.05) contents of protein and concentrations of all measured animo acids (AAs) in eggs from birds fed low AMEn; (5) supplementation with high AMEn to breeder hens significantly increased the hatchability of fertilized eggs; (6) neither dietary lysine level or AMEn affected growth performance of offspring broilers; (7) both dietary lysine level and AMEn significantly affected gonadotropin concentrations and biochemical variables of breeder hens.

**Conclusions:**

Dietary lysine had significant influences on productive performance, reproductive traits, and egg quality of yellow-feathered breeder hens. Based on productive performance, the optimal levels of dietary lysine were 0.81% to 0.83%, while 0.71% to 72% lysine was enough to obtain the best quality of breeding eggs. High AMEn was more beneficial to breeder hens for reproductive traits and hatchability of the fertilized eggs, while it showed detrimental effects on eggshell thickness and AA concentrations of breeding eggs.

## Introduction

In commercial diets (corn-soybean meal based) for broilers, lysine is one of the most limiting essential amino acids (AAs) and necessary for proper growth and development of chickens [1, 2], especially for the turnover of muscle protein where it produces a marked effect in modulating protein biosynthesis and turnover [3]. Energy, is required for the maintenance of body temperature, and synthesis of organic tissues of broilers [4]. Studies have shown the importance of lysine for reproduction, as it promoted the development of reproductive organs and improved production performance of Ross 308 breeder hens [5] and layer hens [6]. Energy is also critical for egg production of layer hens [4, 7] and higher energy improved productive traits in Ross 308 breeder hens [8]. However, so far, research only showed the effects of lysine or AMEn on productive performance of commercial white-feathered broiler breeder hens, with a lack of studies on yellow-feathered broiler breeder hens. The effects of lysine or AMEn on egg quality of layers were also studied, while there is few study exploring the effects on the hatching performance of eggs from breeder hens, and the growth performance of offspring broilers.

Yellow-feathered broilers provide consumers with high-quality meat products and play an important role in poultry production worldwide [9]. In China, the annual sales of yellow-feathered broilers are approximately four billion birds, comparable to white-feathered broilers [10]. Based on Nutrient Requirements of Yellow Chickens (2020) [11], the recommended dietary lysine for broiler breeder hens was 0.68% to 0.78%. There was 0.56% lysine in the basal diet used in this research (without L-lysine supplementation), so 5 levels of lysine (0.56%, 0.68%, 0.80%, 0.92%, and 1.04%) were used. Dietary energy is closely related to the development of sexual organs, and higher energy increased weight of sexual organs, thus improved the productive performance of broiler breeder pullets [8], so the recommended AMEn (11.10 MJ/kg, 2,650 kcal/kg) from Nutrient Requirements of Yellow Chickens [11] and a higher one (11.50 MJ/kg, 2,750 kcal/kg) were used in this research. The productive performance, reproductive traits, biochemical variables of breeder hens, the concentrations of amino acid, quality, and hatching performance of eggs, and the growth performance of offspring broilers were determined to clear explain the effects of lysine and AMEn on breeder hens, and to obtain the optimal nutrient levels for yellow-feathered breeder hens. The results will provide guidance for practical production of yellow-feathered breeder hens.

## Materials and methods

### Experimental design and chicken husbandry

The present study used a 5 × 2 factorial arrangement of treatments to assess main effects and interaction of dietary lysine (0.56%, 0.68%, 0.80%, 0.92%, and 1.04%) and AMEn (11.50 and 11.10 MJ/kg) in yellow-feathered breeder hens (Table 1). A total of 720 30-week-old breeder hens (2.55 ± 0.18 kg) with similar laying rates were used. Birds were randomly allocated to the 10 treatments and received diets supplemented with incremental levels of lysine and AMEn for 12 weeks. Each treatment contained 6 replicates with 12 birds per replicate. The breeders were housed in laying cages (325 mm × 450 mm × 410 mm, 2 birds/cage), and provided with 120 g/d of feed per bird, and access to fresh water ad libitum. Birds were exposed to 16 h light daily.

**Table 1:** Experimental design

Diets	Lys, %	AMEn, MJ/kg
I	0.56	11.50
II	0.68	11.50
III	0.80	11.50
IV	0.92	11.50
V	1.04	11.50
VI	0.56	11.10
VII	0.68	11.10
VIII	0.80	11.10
IX	0.92	11.10
X	1.04	11.10

During the last 2 weeks, each breeder received artificial insemination every 3 d (25 μL, pooled semen). At the end of the trial, 20 eggs per replicate were incubated [12]. Fifteen hatched chicks per replicate were raised with the same diets for 84 d. The rearing and husbandry of offspring broilers was as already described [13].

### Experimental diets

The basal diets were formulated according to the recommended nutrition of breeder hens and nutritive values of feedstuffs in Nutrient Requirements of Yellow Chickens [11]. The details of basal diets for breeder hens and offspring broilers are provided in Tables 2 and 3, respectively. The crude protein of diets was determined according to the Association of Official Analytical Chemists [14]. Lysine level in diets was determined using the hydrolysis and extraction method [15]. In brief, proteins in feed were hydrolyzed into AAs in hydrochloric acid solution at 110 °C, separated by ion exchange chromatography and derivatized by ninhydrin column, and then determined at a wavelength of 570 nm with AA analyzer (L-8900, Hitachi High-Technologies, Tokyo, Japan).

**Table 2:** Composition and nutrient content of diets for broiler breeder hens (as-fed basis)

Diets	I	II	III	IV	V	VI	VII	VIII	IX	X
Ingredients, %										
Corn	61.39	61.39	61.39	61.39	61.39	55.83	55.83	55.83	55.83	55.83
Wheat	7.85	7.85	7.85	7.85	7.85	14.42	14.42	14.42	14.42	14.42
Corn gluten meal (CP 7.8%)	7.75	7.75	7.75	7.75	7.75	7.40	7.40	7.40	7.40	7.40
Soybean meal (CP 48.0%)	10.50	10.50	10.50	10.50	10.50	9.88	9.88	9.88	9.88	9.88
Soybean oil	1.00	1.00	1.00	1.00	1.00	1.00	1.00	1.00	1.00	1.00
Limestone	7.00	7.00	7.00	7.00	7.00	7.00	7.00	7.00	7.00	7.00
Dicalcium phosphate	2.05	2.05	2.05	2.05	2.05	2.00	2.00	2.00	2.00	2.00
NaCl	0.25	0.25	0.25	0.25	0.25	0.24	0.24	0.24	0.24	0.24
DL-Met (99%)	0.14	0.14	0.14	0.14	0.14	0.15	0.15	0.15	0.15	0.15
L-Trp (99%)	0.04	0.04	0.04	0.04	0.04	0.04	0.04	0.04	0.04	0.04
L-Ile (99%)	0.03	0.03	0.03	0.03	0.03	0.04	0.04	0.04	0.04	0.04
L-Lys HCl (78%)	0.00	0.15	0.30	0.46	0.61	0.00	0.15	0.30	0.46	0.61
Zeolite powder	1.00	0.85	0.70	0.54	0.39	1.00	0.85	0.70	0.54	0.39
Vitamin and mineral premix^1^	1.00	1.00	1.00	1.00	1.00	1.00	1.00	1.00	1.00	1.00
Total	100.00	100.00	100.00	100.00	100.00	100.00	100.00	100.00	100.00	100.00
Nutrient contents^2^										
AME, kcal/kg	11.50	11.50	11.50	11.50	11.50	11.10	11.10	11.10	11.10	11.10
CP^3^, %	15.64	15.97	15.35	15.57	16.09	15.80	16.29	16.51	15.93	16.14
Ca, %	3.00	3.00	3.00	3.00	3.00	3.00	3.00	3.00	3.00	3.00
P, %	0.74	0.74	0.74	0.74	0.74	0.74	0.74	0.74	0.74	0.74
Non-phytate phosphorus, %	0.45	0.45	0.45	0.45	0.45	0.45	0.45	0.45	0.45	0.45
Lysine, %	0.56	0.68	0.80	0.92	1.04	0.56	0.68	0.80	0.92	1.04
Lysine^3^, %	0.54	0.67	0.83	0.94	1.05	0.58	0.71	0.80	0.94	1.07
Methionine, %	0.42	0.42	0.42	0.42	0.42	0.42	0.42	0.42	0.42	0.42
Met + Cys %	0.68	0.68	0.68	0.68	0.68	0.68	0.68	0.68	0.68	0.68

^1^Premix provided the following per kilogram of diets: vitamin A, 15,000 IU; vitamin D_3_, 2,160 IU; vitamin E, 27 IU; vitamin K, 1.4 mg; riboflavin, 8.0 mg; nicacin, 32 mg; pantothenic acid, 11 mg; vitamin B_12_, 0.01 mg; biotin, 0.18 mg; folic acid, 1.08 mg; vitamin B_1_, 1.8 mg; vitamin B_6_, 4.1 mg; Cu, 7.0 mg; Zn, 72 mg; Mn, 90 mg; Fe, 72 mg; I, 0.90 mg; Se, 0.27 mg

^2^Values were calculated from data provided by Nutrient Requirements of Yellow Chickens [11]

^3^Values were determined by analysis [14, 15]

**Table 3:** Composition and calculated nutrient content of diets for offspring broilers (as-fed basis)

Growth stage	d 1 to 28	d 29 to 56	d 57 to 84
Ingredients, %			
Corn	61.93	64.40	68.40
Fishmeal	2.00	1.00	0.00
Corn gluten meal (CP 7.8%)	2.00	3.00	4.00
Soybean (CP 48.0%)	28.40	23.60	16.15
Soybean oil	1.00	2.50	4.00
Limestone	0.00	0.10	0.25
Calcium monohydrogen phosphate	0.18	0.11	0.13
NaCl	0.00	0.00	0.02
L-Lys HCl (78%)	0.00	0.01	0.10
DL-Met (99%)	1.20	1.10	1.04
Ile (98%)	1.70	1.60	1.55
L-Thr (99%)	0.09	0.10	0.11
Zeolite powder	0.50	1.48	3.25
Vitamin and mineral premix^1^	1.00	1.00	1.00
Total	100.00	100.00	100.00
Nutrient contents^2^			
AMEn, MJ/kg	11.50	11.50	11.50
CP^3^, %	20.52	18.18	16.39
Ca, %	1.00	0.90	0.81
P, %	0.72	0.66	0.59
Non-phytate phosphorus, %	0.45	0.40	0.36
Lysine, %	1.07	0.98	0.86
Lysine^3^, %	1.20	1.01	0.80
Methionine, %	0.52	0.42	0.40
Met + Cys %	0.85	0.73	0.66

^1^Premix provided the following per kilogram of diets during 1 to 28 days of age: VA 15,000 IU, VD_3_ 3,300 IU, VE 20 IU, VK_3_ 6 mg, VB_1_ 1.8 mg, VB_2_ 9 mg, VB_6_ 3.5 mg, VB_12_ 0.01 mg, choline 500 mg, niacin 60 mg, D-pantothenic acid 16 mg, folic acid 0.55 mg, biotin 0.15 mg, Fe 80 mg, Mn 80 mg, Cu 8 mg, Zn 60 mg, I 0.35 mg, Se 0.3 mg; Premix provided the following per kilogram of diets during 29 to 56 days of age: VA 15,000 IU, VD_3_ 3,300 IU, VE 20 IU, VK_3_ 6.0 mg, VB_1_ 3.0 mg, VB_2_ 9.0 mg, VB_6_ 6.0 mg, VB_12_ 0.03 mg, choline 1,000 mg, niacin 60 mg, D-pantothenic acid 18 mg, folic acid 0.75 mg, biotin 0.10 mg, Fe 80 mg, Mn 100 mg, Cu 12 mg, Zn 75 mg, I 0.35 mg, Se 0.15 mg; Premix provided the following per kilogram of diets during 57 to 84 days of age: VA 10,000 IU, VD_3_ 1,000 IU, VE 20 IU, VK_3_ 4 mg, VB_1_ 1.8 mg, VB_2_ 8 mg, VB_6_ 3.5 mg, VB_12_ 0.01 mg, choline 500 mg, niacin 44 mg, D-pantothenic acid 10 mg, folic acid 0.55 mg, biotin 0.15 mg, Fe 80 mg, Mn 80 mg, Cu 8 mg, Zn 60 mg, I 0.35 mg, Se 0.15 mg

^2^Vaules were calculated from data provided by Nutrient Requirements of Yellow Chickens [11]

^3^Values were determined by analysis [14, 15]

### Measurement of reproductive performance

At the end of the trial, the body weight (BW) of breeder hens was recorded. Eggs were counted and weighed every day, and daily egg mass per replicate was calculated. The unqualified eggs (excessively large or small, misshapen, dirty, with sand shell or without a shell) were recorded. The variables of reproductive performance were calculated using the formulas:

$$\text{Laying rate}\;(\%) =\text{ daily numbers of eggs}/\text{numbers of hens on hand }\times 100\%$$

$$\text{Feed to egg mass ratio }=\text{ average daily feed intake (g)}/\text{average daily egg mass (g) }$$

$$\text{Rate of qualified eggs}\;(\%) =\text{ numbers of qualified eggs}/\text{numbers of total eggs}\times 100\%$$

### Sample collection

Two breeder hens of each replicate were chosen at the end of the trial and blood was collected in evacuated EDTA-K2 tubes from the wing vein. Plasma was then obtained by centrifuging (1,000 × *g*, 15 min, 4 °C). These 2 birds were then electrically stunned and exsanguinated. The carcass was dissected. The ovaries and oviducts including the infundibulum, enlargement, isthmus, and uterus were removed. The ovarian stroma, remaining after dissection of large and small yellow follicles, was collected and snap-frozen in liquid nitrogen. When used for the determination of biochemical variables, the ovarian stroma was homogenized with ice-cold PBS (1:10, v/v), and clarified supernatant was obtained after centrifuging (2,000 × *g*, 10 min).

### Analysis of reproductive traits

The weight of oviduct was measured. The large yellow follicles (LYF, > 8 mm) and small yellow follicles (SYF, 3–8 mm) were identified and counted by measuring the diameter with a Vernier caliper. The ovarian stroma, oviduct, LYF, and SYF were weighed, respectively. The relative weight of these organs or tissues relative to BW was calculated as follows:

$$\text{Relative weight}=\text{weight}/\text{BW}\times100\%$$

### Determinations of egg quality

At 10^th^ week of the study, 3 eggs/replicate (close to the average egg weight) were chosen to determine the egg quality. The major and minor axises were measured with a micrometer. An automatic egg analyzer (EMT-5200 Robotmation Co., Ltd., Tokyo, Japan) was used to determine the yolk color, albumen height, and Haugh unit. An eggshell strength tester (FGV-10XY, Orka Food Technology, Ramat HaSharon, Israel) was used to measure eggshell strength. The yolk was weighed, and eggshell thickness was measured with a micrometer. The relative yolk weight to egg weight and the shape index of egg was calculated using the formulas:

$$\text{Relative yolk weight}\;(\%) =\text{ yolk weight}/\text{egg weight }\times 100\%$$

$$\text{Shape index }=\text{ major axis }(\text{mm}) /\text{minor axis of egg }(\text{mm})$$

### Measurement of hatching quality

At hatch, the weight of chicks was recorded, and the hatching weight of chick/egg weight was calculated.

$$\text{Fertilization rate}\;(\%) = (\text{number of eggs incubated }-\text{ number of unfertilized eggs}) /\text{number of eggs incubated }\times 100\%$$

$$\text{Hatchability}\;(\%) = (\text{number of fertilized eggs }-\text{ number of hatched eggs}) /\text{number of fertilized eggs }\times 100\%$$

### Determinations of amino acid concentrations in eggs

At the end of the trial, for birds treated with 0.56%, 0.80%, and 1.04% lysine (treatments I, III, V, VI, VIII, and X), one egg per replicate was selected to determine concentrations of AAs. After removing eggshell, the yolk and albumen were together weighed and freeze-dried. Crude protein and dry matter were analyzed according to the Association of Official Analytical Chemists [14]. Quantitative analysis of AAs in eggs was performed using the hydrolysis and extraction method, with subsequent readings via HPLC by Guangzhou KingMed Diagnostics Group Co., Ltd. (Guangzhou, China).

### Measurement of growth performance of offspring broilers

The growth performance of the offspring from each replicate was determined. Birds were weighed at 1 and 84 days of age. Dead birds were recorded. The final BW, average daily feed intake, average daily gain, feed to gain ratio, and mortality were calculated.

### Determinations of biochemical variables and gonadotropin concentration in breeders

The activities of alkaline phosphatase (AKP), total antioxidant capacity (T-AOC), total superoxidase dismutase (T-SOD), glutamic oxaloacetic transaminase (GOT) and glutamic pyruvic transaminase (GPT), and the concentration of uric acid (UA), malondialdehyde (MDA), total protein (TP), and albumin (ALB) in the plasma, along with the activities of T-AOC and T-SOD and the concentration of MDA in ovarian stroma were analyzed spectrophotometrically using commercial kits (A059-1-1, A015-1-2, A001-1-2, C010-1-1, C009-1-1, C012-1-1, A003-1-2, A045-2-2 and A028-1-1, Nanjing Jiancheng Bioengineering Institute, Nanjing, China) and a spectrophotometer (Biomate 5, Thermo Electron Corporation, Rochester, NY, USA). The level of follicle stimulating hormone (FSH) in plasma was determined with ELISA kits (Jiangsu Meimian Industrial Co., Ltd., Jiangsu, China) and the above spectrophotometer.

### Statistical analysis

The main effects (lysine and AMEn) and interactions between them were examined by multivariate analysis of variance using SPSS 17.0 for Windows. Means were compared by Duncan multiple range tests at *P* < 0.05 significance levels. Furthermore, in the case of a significant effect (*P* < 0.05) of lysine × AMEn interaction, the linear and quadratic effect of lysine × AMEn were also analyzed to better describe the cause of the interaction (SAS 8.1) [16].

Where appropriate, polynomial regressions were fitted to test for linear and quadratic effects in response to lysine. When a significant quadratic component was demonstrated (*P* < 0.05), optimal dietary lysine was estimated as the lower level achieving 95% of the maximal or minimal response. When an interaction is significant, analysis of polynomial regressions was conducted separately for two AMEn levels.

## Results

### Productive performance

As shown in Table 4, dietary lysine had quadratic effects (*P* < 0.05) on laying rate, average daily egg mass and feed intake/egg mass of yellow-feathered breeder hens. The optimal levels of dietary lysine estimated from the quadratic regressions were 0.83%, 0.82%, and 0.81% for laying rate, average daily egg mass, and feed intake/egg mass, respectively.

**Table 4:** Effects of dietary lysine and AMEn on productive performance of yellow-feathered broiler breeders^1^

Treatment		Final body weight, kg	Laying rate, %	Average daily egg mass, g	Feed intake/egg mass	Average egg weight, g	Rate of qualified eggs, %
**Lys, %**	**AMEn, MJ/kg**						
0.56	11.50	2.86	53.62	32.84	3.61	61.69	95.95
0.68	11.50	2.82	56.53	34.80	3.40	61.68	94.89
0.80	11.50	2.90	58.90	36.15	3.27	61.79	97.10
0.92	11.50	2.92	57.27	35.73	3.31	62.77	94.84
1.04	11.50	2.95	55.02	34.96	3.39	60.96	96.80
0.56	11.10	2.81	54.35	33.30	3.55	61.44	96.87
0.68	11.10	2.66	57.29	35.04	3.36	61.66	95.75
0.80	11.10	2.67	59.37	35.89	3.29	60.72	96.72
0.92	11.10	2.76	56.92	34.79	3.40	61.79	95.74
1.04	11.10	2.62	54.31	32.06	3.70	61.36	95.29
SEM		0.11	1.43	0.89	0.68	0.87	0.09
0.56		2.84	53.98^b^	33.07^b^	3.58^a^	61.56	96.41
0.68		2.74	56.91^a^	34.92^a^	3.38^b^	61.67	95.32
0.80		2.89	59.13^a^	36.02^a^	3.28^b^	61.39	96.86
0.92		2.84	57.10^b^	35.26^a^	3.36^b^	62.27	95.33
1.04		2.79	53.77^c^	32.96^b^	3.60^a^	61.68	96.35
SEM		0.07	1.01	0.63	0.48	1.03	0.06
	11.10	2.71^b^	56.09	34.22	3.46	61.40^b^	96.04
	11.50	2.89^a^	56.27	34.68	3.42	61.96^a^	96.07
	SEM	0.04	0.64	0.40	0.30	0.64	0.04
Mean effects							
Lys^2^		0.902	0.001	0.002	0.001	0.344	0.098
Linear		0.760	0.449	0.289	0.359	0.865	0.803
Quadratic		0.953	< 0.001	0.001	0.001	0.736	0.441
AMEn		0.001	0.833	0.388	0.445	0.043	0.961
Lys × AMEn		0.058	0.763	0.694	0.630	0.701	0.130
Linear Lys × AMEn		0.195	0.534	0.047	0.039	0.869	0.102
Quadratic Lys × AMEn		0.969	0.855	0.422	0.384	0.177	0.530

Quadratic regression equations based on supplemental lysine level (%):

(a) Laying rate (%) = −56.96 lysine^2^ + 94.03 lysine + 19.34, *P* = 0.020, *R*^2^ = 0.783, which yielded optimized lysine level as 0.83%

(b) Average daily egg mass (g) = −674.17 lysine^2^ + 1,102.9 lysine + 122.54, *P* = 0.003, *R*^2^ = 0.987, which yielded optimized lysine level as 0.82%

(c) Feed intake/egg mass = 4.45 lysine^2^−7.25 lysine + 6.24, *P* = 0.001, *R*^2^ = 0.995, which yielded optimized lysine level as 0.81%

*Lys* Lysine, *AMEn* Nitrogen-corrected apparent metabolizable energy

^a–c^Within a column, means without common superscripts within a main effect differ significantly (*P* < 0.05)

^1^For the interaction effect, values are means of 6 replicates per treatment each with 16 broilers. For the main effects, values are means of 12 replicates each with 16 broilers or 30 replicates each with 16 broilers, respectively

^2^Linear and quadratic effects of lysine were tested when significant main effect was detected

Dietary AMEn had a significant effect on final BW of breeder hens, and birds with 11.50 MJ/kg AMEn had higher body weight than those with 11.10 MJ/kg AMEn (*P* < 0.05). Furthermore, there was no interaction (*P* > 0.05) between dietary lysine and AMEn on variables of productive performance.

### Reproductive traits

As presented in Table 5, dietary lysine had significant effect on the relative weight of ovarian stroma (quadratic and linear), and numbers of LYF (quadratic). Dietary AMEn had significant influence on the numbers of LYF, and birds with 11.50 MJ/kg AMEn had more LYF than those with 11.10 MJ/kg AMEn (P < 0.05).

**Table 5:** Effects of dietary lysine and AMEn on reproductive traits of yellow-feathered broiler breeders^1^

Treatment		Fallopian tube	Ovarian stroma	LYFs		SYFs	
**Lys, %**	**AMEn, MJ/kg**	**Weight/BW, %**	**Weight/BW, %**	**Number**	**Weight/BW, %**	**Number**	**Weight/BW, %**
0.56	11.50	1.68	0.20	4.50	1.22	14.75	1.61
0.68	11.50	1.83	0.22	4.87	1.45	15.13	1.94
0.80	11.50	1.82	0.24	4.50	1.26	14.63	1.69
0.92	11.50	1.92	0.22	4.88	1.30	14.25	1.71
1.04	11.50	1.74	0.21	4.13	1.34	14.13	1.80
0.56	11.10	1.70	0.18	3.63	1.16	13.88	1.52
0.68	11.10	1.81	0.20	4.25	1.48	16.00	2.01
0.80	11.10	1.90	0.20	4.50	1.42	14.57	1.43
0.92	11.10	1.74	0.20	4.25	1.36	16.25	2.09
1.04	11.10	1.79	0.26	4.50	1.44	11.75	1.35
SEM		1.52	0.02	0.20	0.10	1.35	0.17
Main effect							
0.56		1.69	0.19^c^	4.06^b^	1.19	14.31	1.56^c^
0.68		1.82	0.21^abc^	4.56^a^	1.46	15.56	1.97^a^
0.80		1.89	0.22^ab^	4.54^a^	1.37	14.60	1.63^bc^
0.92		1.83	0.21^bc^	4.56^a^	1.33	15.25	1.90^ab^
1.04		1.77	0.24^a^	4.31^ab^	1.39	12.94	1.57^c^
SEM		1.08	0.01	0.10	0.07	0.96	0.12
	11.10	1.80	0.21	4.24^b^	1.38	14.49	1.70
	11.50	1.80	0.22	4.58^a^	1.31	14.58	1.75
	SEM	0.61	0.01	0.08	0.04	0.54	0.07
Source of variation							
Lys^2^		0.218	0.020	0.012	0.091	0.357	0.024
Linear		0.390	0.006	0.265	0.239	0.315	0.904
Quadratic		0.061	0.053	0.016	0.188	0.182	0.294
AMEn		0.965	0.235	0.002	0.266	0.922	0.654
Lys × AMEn		0.464	0.041	0.001	0.730	0.566	0.136
Linear Lys × AMEn		0.843	0.032	0.001	0.451	0.760	0.581
Quadratic Lys × AMEn		0.691	0.062	0.777	0.586	0.201	0.253

Quadratic regression equations based on supplemental lysine level (%):

(a) When AMEn is 11.50 MJ/kg, Number of LYFs = −7.44lysine^2^ + 11.28lysine + 0.527,* P* = 0.027, *R*^2^ = 0.178, which yielded optimized lysine level as 0.76%; When AMEn is 11.10 MJ/kg, Number of LYFs = −6.20lysine^2^ + 11.379lysine – 0.731,* P* = 0.004, *R*^2^ = 0.258, which yielded optimized lysine level as 0.92%

*Lys* Lysine, *AMEn* Nitrogen-corrected apparent metabolizable energy, *LYFs* Large yellow follicles, follicles with mean diameter > 8 mm, *SYFs* Small yellow follicles, follicles with mean diameter of 3 to 8 mm

^a–c^Means without common superscripts within a main effect differ significantly (*P* < 0.05)

^1^For the interaction effect, values are means of 6 replicates each with 2 broilers. For the main effects, values are means of 12 replicates each with 2 broilers or 40 replicates each with 2 broilers, respectively

^2^When lysine, the treatment effect was significant, linear and quadratic effects were tested

Interactions existed between dietary lysine and AMEn for the relative ovarian weight and numbers of LYF. The optimal levels of dietary lysine estimated. For numbers of LYF, the optimal lysine level was 0.76% when AMEn was 11.50 MJ/kg, and 0.92% when AMEn was 11.10 MJ/kg, respectively.

### Egg quality

Dietary lysine had both linear and quadratic effects (*P* < 0.05) on eggshell thickness and shell strength of eggs from breeder hens (Table 6). Dietary AMEn significantly affected on eggshell thickness and yolk color (*P* < 0.05), and birds with 11.50 MJ/kg AMEn had thinner eggshell and deeper yolk color than those fed 11.10 MJ/kg AMEn (*P* < 0.05).

**Table 6:** Effects of dietary lysine and AMEn on egg quality of yellow-feathered broiler breeders^1^

Treatment		Shape index	Eggshell thickness, mm	Shell strength, kgf	Relative yolk weight, %	Yolk color	Albumen height, mm	Haugh unit
**Lys, %**	**AMEn, MJ/kg**							
0.56	11.50	1.30	3.92	0.335	31.70	10.15	5.33	70.91
0.68	11.50	1.30	4.98	0.359	31.99	10.04	5.43	70.86
0.80	11.50	1.30	4.17	0.344	32.12	9.82	5.42	70.16
0.92	11.50	1.31	3.91	0.323	32.76	9.68	5.95	75.01
1.04	11.50	1.30	3.86	0.321	32.66	9.90	5.68	73.61
0.56	11.10	1.30	4.57	0.340	31.36	9.40	5.88	74.91
0.68	11.10	1.31	4.65	0.345	31.93	9.06	5.03	67.59
0.80	11.10	1.29	4.44	0.341	32.23	9.40	5.23	69.76
0.92	11.10	1.30	4.52	0.341	31.43	9.44	5.34	70.12
1.04	11.10	1.32	4.18	0.333	31.95	9.61	5.32	70.41
SEM		0.01	0.19	0.00	0.36	0.14	0.28	2.45
Main effect								
0.56		1.29	4.32^bc^	0.337^bc^	31.36	9.80	5.73	74.06
0.68		1.30	4.82^a^	0.361^a^	31.68	9.54	5.23	69.28
0.80		1.29	4.45^b^	0.344^ab^	31.92	9.68	5.60	73.25
0.92		1.31	4.33^bc^	0.335^c^	32.09	9.63	5.85	74.64
1.04		1.32	4.04^c^	0.327^d^	32.19	9.78	5.60	73.07
SEM		0.01	0.13	0.00	0.26	0.10	0.19	1.73
	11.10	1.30	4.57^a^	0.34	31.64	9.42^b^	5.49	71.97
	11.50	1.30	4.21^b^	0.34	32.06	9.95^a^	5.71	73.75
	SEM	0.01	0.08	0.00	0.16	0.06	0.12	1.09
Source of variation								
Lys^2^		0.326	< 0.001	< 0.001	0.116	0.130	0.126	0.090
Linear		0.119	0.010	< 0.001	0.045	0.909	0.805	0.745
Quadratic		0.619	0.003	< 0.001	0.062	0.619	0.900	0.845
AMEn		0.656	0.001	0.083	0.064	0.000	0.158	0.181
Lys × AMEn		0.124	0.053	< 0.001	0.101	0.003	0.074	0.155
Linear Lys × AMEn		0.784	0.703	0.027	0.232	0.002	0.104	0.149
Quadratic Lys × AMEn		0.761	0.216	0.118	0.639	0.936	0.228	0.423

Quadratic regression equations based on supplemental lysine level (%):

(1) Eggshell thickness (mm) = −5.57 lysine^2^ + 8.02 lysine + 1.64, *P* = 0.003, *R*^2^ = 0.087, which yielded optimized lysine level as 0.72%

(2) When AMEn is 11.50 MJ/kg, eggshell strength (kgf) = −0.29 lysine^2^ + 0.41 lysine + 0.20, *P* < 0.001, *R*^2^ = 0.305, which yielded optimized lysine level as 0.71%; When AMEn is 11.10 MJ/kg, eggshell strength (kgf) = −0.11 lysine^2^ + 0.16 lysine + 0.29, *P* = 0. 158, *R*^2^ = 0.054

*Lys* Lysine, *AMEn* Nitrogen-corrected apparent metabolizable energy

^a−d^Means within a main effect, without common superscripts differ significantly (*P* < 0.05)

^1^For the interaction effect, values are means of 6 replicates each with 3 eggs each. For the main effects, values are means of 12 replicates each with 3 eggs or 40 replicates each with 3 eggs, respectively

^2^When lysine, the treatment effect was significant, linear and quadratic effects were tested

Interactions were found between lysine and AMEn for eggshell strength (*P* < 0.05) and yolk color (*P* < 0.05). The optimal levels of dietary lysine from the quadratic regressions were 0.71% for eggshell strength (when AMEn was 11.50 MJ/kg), and 0.72% for eggshell thickness.

### Concentrations of amino acids in eggs

As shown in Table 7, there were higher (*P* < 0.05) levels of DM, CP, and concentrations of all measured AAs in eggs from birds fed 11.10 compared to 11.5 MJ/kg AMEn. Dietary lysine only affected concentrations of proline in eggs, where those in eggs from birds fed 0.80% and 1.02% lysine were increased over birds fed 0.56% lysine.

**Table 7:** Effects of dietary lysine and AMEn on the concentrations of amino acid in eggs of yellow-feathered broiler breeders^1^

Treatment		DM	CP	TAA	Ala	Arg	Asp	Glu	Gly	His	Ile	Leu	Lys	Met	Phe	Pro	Ser	Thr	Tyr	Val
**Lys, %**	**AMEn, MJ/kg**																			
0.56	11.50	34.99	16.64	103.82	6.20	7.53	10.67	13.42	3.58	2.70	5.03	9.07	8.57	4.85	5.98	2.17	8.62	4.87	4.60	5.95
0.80	11.50	35.45	15.97	99.42	6.17	7.10	10.23	11.58	3.45	2.52	4.88	8.80	7.93	4.25	5.93	1.85	8.25	4.52	4.38	5.83
1.04	11.50	35.59	17.10	107.00	6.57	7.88	10.87	14.07	3.62	2.82	5.18	9.50	8.83	4.65	6.23	1.88	9.02	4.87	4.82	6.22
0.56	11.10	38.23	17.65	113.47	7.02	8.23	11.50	15.00	3.85	2.98	5.53	10.07	9.45	4.78	6.7	1.90	9.53	5.08	5.12	6.63
0.80	11.10	36.37	17.34	123.67	7.57	8.72	12.45	16.35	4.25	3.22	6.07	10.93	9.87	4.93	7.32	3.33	10.12	5.93	5.48	7.23
1.04	11.10	37.64	17.99	114.17	6.87	7.68	11.57	15.85	3.80	3.02	5.20	9.73	9.37	5.12	6.82	3.20	9.03	5.52	4.85	6.43
SEM		0.89	0.52	4.23	0.26	0.31	0.40	0.97	0.14	0.13	0.22	0.37	0.37	0.22	0.27	0.31	0.36	0.24	0.18	0.26
Main effect																				
0.56		36.61	17.14	108.64	6.61	7.88	11.08	14.21	3.72	2.84	5.28	9.57	9.01	4.82	6.36	2.03^b^	9.08	4.98	4.86	6.29
0.80		35.87	16.59	111.54	6.87	7.91	11.34	13.97	3.85	2.87	5.48	9.87	8.90	4.59	6.63	2.59^a^	9.18	5.23	4.93	6.53
1.04		36.61	17.55	110.58	6.72	7.78	11.22	14.96	3.71	2.92	5.19	9.62	9.10	4.88	6.53	2.54^a^	9.03	5.19	4.83	6.33
SEM		0.63	0.37	2.99	0.18	0.22	0.28	0.68	0.10	0.09	0.16	0.27	0.26	0.16	0.19	0.22	0.25	0.17	0.13	0.18
	11.10	37.46^a^	17.66^a^	117.10^a^	7.15^a^	8.21^a^	11.84^a^	15.73^a^	3.97^a^	3.07^a^	5.60^a^	10.24^a^	9.56^a^	4.94^a^	6.96^a^	2.81^a^	9.56^a^	5.51^a^	5.15^a^	6.77^a^
	11.50	35.33^b^	16.54^b^	103.41^b^	6.31^b^	7.51^b^	10.59^b^	13.02^b^	3.55^b^	2.68^b^	5.03^b^	9.12^b^	8.44^b^	4.58^b^	6.05^b^	1.97^b^	8.63^b^	4.75^b^	4.60^b^	6.00^b^
	SEM	0.52	0.30	2.44	0.15	0.18	0.23	0.56	0.08	0.07	0.13	0.22	0.22	0.13	0.16	0.18	0.21	0.14	0.11	0.15
Source of variation																				
Lys		0.632	0.236	0.574	0.227	0.573	0.514	0.693	0.213	0.928	0.365	0.472	0.781	0.346	0.391	0.006	0.840	0.220	0.689	0.374
AMEn		0.006	0.013	< 0.001	< 0.001	0.001	< 0.001	< 0.001	< 0.001	< 0.001	0.001	< 0.001	< 0.001	0.040	< 0.001	< 0.001	< 0.001	< 0.001	< 0.001	< 0.001
Lys × AMEn		0.381	0.883	0.024	0.027	0.007	0.026	0.053	0.016	0.062	0.024	0.004	0.051	0.191	0.110	< 0.000	0.008	0.002	0.000	0.011
Linear Lys × AMEn		0.450	0.926	0.670	0.155	0.075	0.816	0.896	0.671	0.667	0.150	0.149	0.542	0.205	0.670	< 0.001	0.110	0.165	0.053	0.214
Quadratic Lys × AMEn		0.244	0.627	0.004	0.010	0.003	0.006	0.026	0.003	0.010	0.003	0.002	0.019	0.186	0.041	0.004	0.006	0.001	< 0.001	0.006

*DM* Dry matter, *CP* Crude protein, *TAA* Total amino acids, *AMEn* Nitrogen-corrected apparent metabolizable energy, *Ala* Alanine, *Arg* Arginine, *Asp* Aspartic acid, *Glu* Glutamic acid, *Gly* Glycine, *His* Histidine, *Ile* Isoleucine, *Leu* Leucine, *Lys* Lysine, *Met* Methionine, *Phe* Phenylalanine, *Pro* Proline, *Ser* Serine, *Thr* Threonine, *Tyr* Tyrosine, *Val* Valine

^a,b^Means within a main effect, without common superscripts differ significantly (*P* < 0.05)

^1^For the interaction effect, values are means of 6 replicates per treatment. For the main effects, values are means of 12 replicates per treatment or 18 replicates, respectively

Interactions existed (*P* < 0.05) between dietary lysine and AMEn for the concentrations of total AAs, alanine, arginine, aspartic acid, glycine, isoleucine, leucine, proline, serine, threonine, tyrosine, and valine.

### Hatching performance

Table 8 shows that dietary lysine did not affect the hatching performance of yellow-feathered breeder hens (*P* > 0.05). Dietary AMEn had significant effect on hatchability of fertilized eggs, where birds given 11.50 MJ/kg AMEn out-performed (*P* < 0.05) those fed 11.10 MJ/kg AMEn. Significant interactions (*P* < 0.05) between lysine and AMEn existed for hatchability of fertilized eggs.

**Table 8:** Effects of dietary lysine and AMEn on hatching performance of yellow-feathered broiler breeders^1^

Treatment		Fertilization rate, %	Hatchability of fertilized eggs, %	Birth weight of chick/egg weight
**Lys, %**	**AMEn, MJ/kg**			
0.56	11.50	94.17	96.57	0.70
0.68	11.50	93.00	96.40	0.71
0.80	11.50	95.83	95.65	0.69
0.92	11.50	98.33	98.25	0.71
1.04	11.50	91.67	96.35	0.69
0.56	11.10	94.17	95.42	0.71
0.68	11.10	97.50	96.57	0.70
0.80	11.10	92.00	96.13	0.72
0.92	11.10	94.17	93.09	0.70
1.04	11.10	95.83	87.83	0.71
SEM		1.97	2.47	0.01
Main effect				
0.56		94.17	96.00	0.70
0.68		95.25	96.12	0.71
0.80		93.92	97.27	0.70
0.92		96.67	93.20	0.70
1.04		93.92	93.39	0.70
SEM		1.39	1.75	0.01
	11.10	94.90	93.37^b^	0.71
	11.50	94.67	97.02^a^	0.70
	SEM	0.88	1.11	0.00
Source of variation				
Lys		0.608	0.186	0.969
Linear		0.780	0.139	0.982
Quadratic		0.893	0.204	0.974
AMEn		0.852	0.006	0.387
Lys × AMEn		0.124	0.001	0.153
Linear Lys × AMEn		0.994	0.066	0.465
Quadratic Lys × AMEn		0.132	0.231	0.617

*Lys* Lysine, *AMEn* Nitrogen-corrected apparent metabolizable energy

^a,b^Means within a main effect, without common superscripts differ significantly (*P* < 0.05)

^1^For the interaction effect, values are means of 6 replicates per treatment each with 16 broilers. For the main effects, values are means of 12 replicates each with 16 broilers or 30 replicates each with 16 broilers, respectively

### Growth performance of offspring broilers

Table 9 shows that, neither main effect of dietary lysine level or AMEn, nor interactions between them affected growth performance of offspring broilers (*P* > 0.05).

**Table 9:** Effects of dietary lysine and AMEn on growth performance of offspring broilers from 1 to 84 days of age^1^

Treatment		Initial body weight, g	Final body weight, kg	ADG, g	ADFI, g	F/G	Survival rate, %
**Lys, %**	**AMEn, MJ/kg**						
0.56	11.50	42.71	1.92	34.96	75.48	2.16	98.89
0.68	11.50	42.55	1.95	35.14	75.00	2.14	100.00
0.80	11.50	42.67	1.93	34.49	74.59	2.14	96.67
0.92	11.50	42.66	1.94	35.10	73.81	2.10	100.00
1.04	11.50	42.51	1.94	34.72	73.22	2.11	98.89
0.56	11.10	42.49	1.95	35.14	74.80	2.13	100.00
0.68	11.10	42.42	1.96	35.16	75.51	2.15	98.89
0.80	11.10	42.45	1.98	35.48	75.79	2.14	100.00
0.92	11.10	42.62	1.97	34.43	73.87	2.15	98.89
1.04	11.10	42.66	2.00	34.45	74.26	2.16	98.89
SEM		0.11	0.02	0.39	0.94	0.02	0.89
Main effect							
0.56		42.60	1.93	35.05	75.14	2.14	99.44
0.68		42.48	1.95	35.15	75.26	2.14	99.44
0.80		42.62	1.95	35.11	75.02	2.14	98.33
0.92		42.64	1.96	34.77	73.84	2.13	99.44
1.04		42.58	1.97	34.59	73.74	2.13	98.89
SEM		0.08	0.01	0.27	0.66	0.01	0.63
	11.10	42.53	1.97	34.93	74.85	2.14	99.33
	11.50	42.64	1.93	34.93	74.35	2.13	98.89
	SEM	0.05	0.01	0.17	0.42	0.01	0.40
Source of variation							
Lys		0.690	0.541	0.565	0.285	0.822	0.707
Linear		0.666	0.086	0.173	0.062	0.343	0.921
Quadratic		0.801	0.217	0.152	0.152	0.442	0.739
AMEn		0.121	0.010	0.999	0.410	0.223	0.446
Lys × AMEn		0.305	0.783	0.535	0.803	0.157	0.127
Linear Lys × AMEn		0.109	0.480	0.373	0.485	0.017	0.580
Quadratic Lys × AMEn		0.457	0.669	0.519	0.659	0.943	0.825

*Lys* Lysine, *AMEn* Nitrogen-corrected apparent metabolizable energy, *ADG* Average daily gain, *ADFI* Average daily feed intake, *F/G* Feed to gain ratio

^1^For the interaction effect, values are means of 6 replicates per treatment each with 16 broilers. For the main effects, values are means of 12 replicates each with 16 broilers or 30 replicates each with 16 broilers, respectively

### Concentration of gonadotrophins in plasma and biochemical variables in plasma and ovarian stroma

Table 10 shows that dietary lysine affected the concentration of FSH (linear and quadratic, *P* < 0.05) in the plasma of breeder hens. The level of FSH was decreased in birds with 11.10 rather than 11.50 MJ/kg AMEn (*P* < 0.05). In the case of plasmal FSH, significant interactions (*P* < 0.05) existed between dietary lysine and AMEn.

**Table 10:** Effects of dietary lysine and AMEn on hormone concentrations and biochemical variables of yellow-feathered broiler breeders^1^

**Treatment**		**Plasma**										**Ovarian stroma**		
**Lys, %**	**AMEn,** **MJ/kg**	**FSH, IU/L**	**TP, mg/mL**	**ALB, mg/mL**	**AKP, U/mL**	**UA,** **nmol/mL**	**GPT, U/mL**	**GOT, U/mL**	**MDA, nmol/mL**	**T-AOC,** **U/mL**	**T-SOD,** **U/mL**	**MDA, nmol/mL**	**T-AOC,** **U/mL**	**T-SOD, U/mL**
0.56	11.50	0.54	65.68	30.57	7.13	75.78	35.21	61.13	6.85	7.39	114.70	16.80	205.83	169.00
0.68	11.50	0.54	70.08	35.89	7.72	92.37	41.17	67.95	6.21	7.99	99.85	17.79	254.38	120.18
0.80	11.50	0.53	58.05	28.85	7.63	71.22	41.35	52.50	5.32	8.74	112.21	18.94	258.73	190.88
0.92	11.50	0.51	64.10	27.17	7.12	76.53	34.45	43.26	5.25	13.18	123.80	17.29	312.81	224.79
1.04	11.50	0.49	62.13	27.81	6.76	71.76	26.63	44.50	6.60	9.77	108.73	14.41	293.47	210.23
0.56	11.10	0.51	66.01	33.23	5.64	88.49	37.65	46.49	3.42	16.23	96.80	13.46	111.10	139.12
0.68	11.10	0.55	61.39	29.18	5.42	83.03	32.43	49.35	3.87	12.75	111.76	16.07	244.27	205.41
0.80	11.10	0.49	59.44	30.24	4.43	80.18	27.07	54.82	5.14	14.03	130.84	19.22	163.48	214.41
0.92	11.10	0.47	59.91	19.60	5.41	94.64	23.77	77.80	4.84	17.69	135.68	11.49	131.41	207.86
1.04	11.10	0.50	60.36	20.01	4.40	91.04	29.50	62.97	3.54	17.74	129.14	10.84	311.28	195.88
SEM		0.01	2.20	1.98	0.64	5.09	5.12	6.87	0.81	1.58	11.12	2.04	43.15	18.66
Main effect														
0.56		0.52^ab^	65.90^a^	32.05^a^	6.24	82.65^a^	35.42	51.51	5.77	10.88^b^	111.68	13.71^b^	158.46	148.41^b^
0.68		0.54^a^	65.64^ab^	31.63^ab^	6.40	86.90^a^	34.42	53.65	5.89	9.02^bc^	111.82	16.93^ab^	234.41	139.90^b^
0.80		0.51^bc^	57.75^c^	29.36^b^	5.70	70.97^b^	31.35	59.23	5.13	10.90^b^	117.99	20.34^a^	197.85	200.04^a^
0.92		0.49^c^	61.07^bc^	22.96^c^	6.00	82.62^a^	26.85	55.87	4.93	14.81^a^	122.51	15.12^b^	159.02	184.65^a^
1.04		0.50^c^	60.15^c^	23.41^c^	5.75	79.47^ab^	26.39	50.75	4.09	13.83^ab^	131.74	13.17^b^	238.85	192.72^a^
SEM		0.01	1.56	1.40	0.45	3.60	3.62	4.86	0.57	1.11	7.87	1.44	30.52	13.19
	11.10	0.50^b^	61.03	26.15^b^	4.99^b^	87.52^a^	29.79	55.58	4.00^b^	15.69^a^	121.07	14.13^b^	150.63^b^	167.96
	11.50	0.53^a^	63.18	29.61^a^	7.05^a^	73.52^b^	31.98	52.82	6.32^a^	8.09^b^	117.22	17.58^a^	244.80^a^	178.32
	SEM	0.01	0.99	0.88	0.29	2.28	2.29	3.07	0.36	0.70	4.98	0.91	19.30	8.34
Source of variation														
Lys^2^		< 0.001	< 0.001	< 0.001	0.799	0.018	0.211	0.281	0.103	< 0.001	0.403	0.009	0.082	< 0.001
Linear		< 0.001	0.007	< 0.001	0.717	0.022	0.004	0.563	0.161	0.033	0.065	0.346	0.208	< 0.001
Quadratic		0.001	0.011	< 0.001	0.585	0.024	0.017	0.247	0.340	0.077	0.170	0.040	0.430	< 0.001
AMEn		0.005	0.098	< 0.001	< 0.001	< 0.001	0.477	0.307	< 0.001	< 0.001	0.596	0.017	0.001	0.117
Lys × AMEn		0.014	0.731	< 0.001	0.660	0.134	0.264	< 0.001	0.007	0.110	0.259	0.601	0.627	< 0.001
Linear Lys × AMEn		0.507	0.991	< 0.001	0.637	0.065	0.885	< 0.001	0.414	0.702	0.149	0.758	0.946	0.379
Quadratic Lys × AMEn		0.194	0.422	0.682	0.378	0.117	0.041	0.789	0.028	0.033	0.386	0.486	0.384	0.023

*Lys* Lysine, *AMEn* Nitrogen-corrected apparent metabolizable energy, *TP* Total protein, *ALB* Albumin, *AKP* Alkaline phosphatase, *UA* Uric acid, *T-SOD* Total superoxide dismutase, *T-AOC* Total antioxidant capability, *MDA* Malondialdehyde, *GOT* Aspartate aminotransferase, *GPT* Alanine aminotransferase, *FSH* Follicle-stimulating hormone

^a–c^Means within a main effect, without common superscripts differ significantly (*P* < 0.05)

^1^For the interaction effect, values are means of 6 replicates per treatment each with 2 breeders. For the main effects, values are means of 12 replicates each with 2 breeders or 30 replicates each with 2 breeders, respectively

^2^When lysine, the treatment effect was significant, linear and quadratic effects were tested

Lysine significantly affected on the levels of TP (quadratic and linea), ALB (quadratic and linear), UA, the T-AOC activity (linear) in plasma, and the MDA level (quadratic) and T-SOD activity (quadratic and linear) in ovarian stroma. The levels of ALB, MDA and the activity of AKP in plasma, the level of MDA and the activity of T-AOC in ovarian stroma were increased. The level of UA and the activity of T-AOC in plasma were decreased in birds with 11.50 MJ/kg AMEn rather than those with 11.10 MJ/kg AMEn (*P* < 0.05). Interactions existed (*P* < 0.05) between lysine and AMEn for the levels of ALB and MDA in plasma, and the level of MDA and the activity of T-SOD in ovarian stroma.

## Discussion

### Effect and interaction of dietary lysine and AMEn on productive performance and reproductive traits of yellow-feathered breeder hens

Lysine is required for protein synthesis [17], thus is essential for the growth and reproduction of chickens. Our research showed that dietary lysine had quadratic effects on laying rate, average daily egg mass and feed intake/egg mass of breeder hens. Previous study showed that, to a certain extent, high dietary lysine increased laying rate, and settable egg production of Ross 308 breeder hens [4]. There was not much research on breeder hens, but for Lohmann Brown layer hens, egg production and egg mass were decreased and FCR was increased when dietary lysine levels were higher than 0.9% [6]. In research with laying ducks, egg production and egg weight were increased linearly and quadratically as dietary lysine levels increased [18]. These together with the current study suggested that excessive or insufficient lysine has adverse effects on productive performance of breeder hens. For yellow-feathered breeder hens, the optimal lysine levels for productive performance were estimated to be 0.81% to 0.83%, which were higher than the requirement for egg production of white-feathered breeder hens [5] (0.71% to 0.77%, Ross 308), and also higher than the recommendation of Nutrient Requirements of Yellow Chickens (0.68% to 0.78%) [11].

In the current study, different lysine levels affected the egg production with no influence on BW of breeder hens, the same with research on Ross 308 breeder hens [5]. Joseph et al. [19] founded that there was no difference on BW of Cobb 500 broiler breeders, but egg production in responses to crude protein level was observed, and Mohiti-Asli et al. [20] suggested that the BW of matured hens might be more susceptible to energy intake. Part of the energy received is directedly used for fat deposition and BW gain [21], thus dietary AMEn has a significant effect on final BW of breeder hens. The current research suggested that birds given high AMEn (11.50 MJ/kg) had higher BW than those provided with low AMEn (11.10 MJ/kg). Similarly, Lu et al. [7] found that the BW of laying breeders decreased linearly with increasing energy restriction; geese received a high ME diet caused an increased weight in liver and abdominal fat [12]. It is worth noting that, in diets with low AMEn, there was more wheat bran used (as Table 2 shown), indicating that fiber might partly contributed to the difference in BW, as Mohiti-Asli et al. [20] found that dietary fiber decreased the BW gain and abdominal fat weight of broiler breeder hens.

Dietary energy is closely related to the development of sexual organs. Previous research found that energy-restricted feeding delayed development of sexual organs, including the number of LYF, oviductal length, and relative weight of ovarian stroma and fallopian tube, and delayed sexual maturity [7, 22]. Feed restriction slowed the growth rate of the follicle and fallopian tube, thereby constrained development of sexual organs of breeder hens [23]. Higher energy increased ovarian weight of Ross 308 broiler breeder pullets, presumably because it partitioned extra nutrients for ovarian development [8]. In the present research, high AMEn increased the numbers of LYF. Birds with high AMEn also had heavier ovarian stroma, but the impact was not significant, and the difference between the current and previous result of white-feathered breeders [8] might be due to the difference in the breed or age of birds (the birds used in the current research were in higher level of sexual maturity, which might lead to less sensitive of sexual development to nutrition). Overall, high energy promoted the development of sexual organs of yellow-feathered breeder hens, and the possible mechanism was higher energy advanced activation of the hypothalamo–pituitary–gonadal axis and stimulated reproductive gonadotropin levels [8].

Research showed that diets with lower lysine had detrimental nutritional consequences resulting from imbalanced dietary AAs and impaired growth of the reproductive organs, the relative weight of fallopian tube and ovary were lower in Ross 308 parent stock hens given 0.59% lysine compared to hens with higher lysine (0.63% to 0.79%) [4]. However, the highest lysine level (0.79%) used in this research was close to the calculated lysine requirement based on linear- and quadratic-plateau models (0.71% to 0.77%), so it was unable to know whether there were adverse effects of excessive lysine on reproductive performance. In the current manuscript, dietary lysine significantly affected the relative weight of ovarian stroma, and numbers of LYF in a quadratic manner, suggesting that excessive or insufficient lysine had adverse effects on reproductive traits of breeder hens. Tian et al. [24] showed that excessive lysine reduced the expression of lipid metabolism genes (*FABP1*, *ACC*, *ME*, *SREBP-1c*, and *PPARα*) in the liver, thus inhibited lipogenesis and eventually led to the reduced abdominal fat deposition in broilers. Lipid synthesis and metabolism are related to energy, which might be the mechanism by which excessive lysine affected reproductive traits of breeder hens. In the current research, interactions existed between dietary lysine and AMEn for the reproductive traits, and the optimal lysine level was higher (0.92%) when birds were given lower energy than those given higher energy (0.76%).

### Effect and interaction of dietary lysine and AMEn on egg quality and their concentrations of amino acids

Dietary lysine had both linear and quadratic effects on eggshell thickness and strength of eggs from breeder hens. Similarly, eggshell strength increased with increases in the dietary lysine for laying hens [25], consistent with other earlier results [26, 27]. The shell matrix containing 70% protein [25], might be affected by protein synthesis and optimal AA composition. For yellow-feathered breeder hens, the optimal level of dietary lysine for eggshell quality was estimated as being 0.71% to 0.72%.

In the current study, high AMEn positively influenced yolk color. The same result was shown in the research of Han et al. [28] in laying hens, likely associated with inclusion of specific feed ingredients for producing different dietary AMEn concentrations. The color of egg yolk directly reflects concentrations of carotenoids in diet that are contained at high amounts in corn or corn-derived ingredients [28–30]. In the current experiment, more corn was used to produce the high-AME diet. Granghelli et al. [4] showed that increasing dietary ME levels increased the eggshell thickness, and Han et al. [28] suggested that normal-energy diet increased eggshell thickness compared with low-energy diet. Interestingly, the current research showed different results. Eggshell biomineralization was regulated by calcium homeostasis in hens, in turn affected by vitamin D metabolites [31] and human researches indicated that the vitamin D deficiency was associated with obesity [32, 33]. Thus, the decline in eggshell thickness might be the consequence of vitamin D decline, which was related to the more energy intake. More researches are needed to explore the specific mechanism how energy regulates the eggshell.

The nutritional needs for embryonic development of birds are obtained from predetermined nutrients in the eggs [34], but there have been only few studies on the deposition of AAs in the egg yolk from breeder hens. Dietary lysine quadratically increased the concentrations of proline, ﻿probably because higher proline was associated to increased protein synthesis [35], and the reduced concentration of proline caused by the lysine-restricted diet might suggest reduced protein anabolism [36]. High dietary AMEn decreased the concentrations of all AAs in eggs, which might be related with the reduced total protein. This finding might be the consequence of the increased fat in egg caused by high dietary AMEn, as more energy would be used for fat deposition of breeder hens [21], and maternal fat might affect the deposition of fat in eggs.

### Effect and interaction of dietary lysine and AMEn on hatching performance of eggs and growth performance of offspring

There is increasing awareness of the importance of maternal diet to offspring health [37]. During the formation of eggs, maternal nutrients are deposited in the eggs and subsequently used by the embryo [38]. There is hence an important connection between maternal nutrition and the growth, development, and normal metabolism of embryos and offspring chickens [13, 39]. In the current study, dietary AMEn had a significant effect on hatchability of the fertilized eggs. Similarly, Enting et al. [40] also found breeder hens that received more energy showed higher hatchability of their fertilized eggs. Firman et al. [41] also found that providing with creatine (a naturally occurring energy source) in ovo also increased the hatchability of eggs, with potentially higher energy reserved available for embryonic growth and hatching.

Maternal dietary lysine level or AMEn and their interactions did not affect any variables related to growth performance of offspring broilers, concurred with previous work that did not find any influence of maternal diet on feed conversion ratio of the offspring [37], that might because numerous environmental and nutritional factors such as egg storage, hatching conditions, rearing conditions, feed quality, and pathogen exposure might additively or synergistically mask the effect of maternal nutrition on the broiler [37].

### Effect and interaction of dietary lysine and AMEn on biochemical variables of yellow-feathered breeder hens

In the present study, dietary lysine increased the activities of T-AOC in plasma and T-SOD in ovarian stroma. Previous studies showed the influence of lysine on strengthening antioxidant capacity in animals, as it markedly improved the activities of antioxidant enzymes such as catalase and glutathione peroxidase in serum and renal tissue of rats [42] and intestine of grass carp [43] via activating the target of rapamycin (TOR) signaling pathway, and suppressing the p38 mitogen-activated protein kinase (p38-MAPK) signaling pathway [43]. The current research also suggested that low lysine increased the TP and ALB in plasma of breeder hens, which probably resulted from lysine restriction leading to altered protein metabolism, mainly increased catabolism [2, 36].

High AMEn decreased the plasmal concentration of MDA and ovarian stroma and increased the activities of AKP and T-AOC. The decline in antioxidant capacity might be related to the weight gain of breeder hens, because diets with high energy caused increased weight of liver and abdominal fat [21, 22] as already discussed. Moreover, high AMEn led to the enhancement of FSH secretion of breeder hens, an important regulator of ovarian function, especially in the recruitment of follicles [44]. Research on mammals showed that the expression level of *FSHR* (follicle stimulating hormone receptor) in sheep with high energy was higher than those with low energy [45]. Higher energy advances the activation of the hypothalamo–pituitary–gonadal axis, stimulated reproductive gonadotropin levels, and upregulated the expression of their receptor genes.

## Conclusion

Dietary lysine had significant influences on productive performance, reproductive traits, and egg quality of yellow-feathered breeder hens. Based on laying rate, average daily egg mass, and feed intake/egg mass, the optimal levels of dietary lysine, estimated from the quadratic regressions, were 0.81% to 0.83%, which were higher than the requirement for white-feathered breeder hens, and also higher than the recommendation of Nutrient Requirements of Yellow Chickens. Levels of 0.71% to 72% was enough obtain the best quality of breeding eggs. In this experiment, compared with low AMEn (11.10 MJ/kg), high AMEn (11.50 MJ/kg) was more beneficial to breeder hens for reproductive traits and hatchability of the fertilized eggs, while it showed detrimental effects on antioxidant capacity of hens and eggshell thickness and AA concentrations of breeding eggs. Furthermore, interactions existed between dietary lysine and AMEn for reproductive traits and egg quality, indicating that lysine requirements should be conducted for different levels of AMEn.

## Data Availability

All data generated or analyzed during this study are available from the corresponding author on request.

## Abbreviations

AA: Amino acid
AKP: Alkaline phosphatase
ALB: Albumin
AMEn: Apparent nitrogen-corrected metabolizable energy
BW: Body weight
FSH: Follicle stimulating hormone
GOT: Glutamic oxaloacetic transaminase
GPT: Glutamic pyruvic transaminase
LYF: The large yellow follicles (> 8 mm)
MDA: Malondialdehyde
SYF: Small yellow follicles (3−8 mm)
T-AOC: Total antioxidant capacity
TP: Total protein
T-SOD: Total superoxidase dismutase
UA: Concentration of uric acid
